# Craniofacial pain and anatomical abnormalities of the nasal cavities

**DOI:** 10.1016/S1808-8694(15)31211-8

**Published:** 2015-10-20

**Authors:** Jeferson Cedaro de Mendonça, Ivo Bussoloti Filho

**Affiliations:** 1 Master studies, physician.; 2 Ph.D. in Otorhinolaryngology, UNIFESP.

**Keywords:** facial, cranio, pain, anatomic, abnormality

## Abstract

The causal relation between anatomical variations of the nose and headaches and facial pain is analyzed through literature review of the topic. The pathogenesis that can be involved in this relation proves to be wider than simple alteration of nasal septum and turbinates that can cause mechanical stimulus through contact between these structures, which covers infectious factors, neurogenic inflammation, correlation with migraines and the role of nasal obstruction. The clinical findings of a lot of authors including the test with topical anesthetic to prove this causal relation, the indication of surgical treatment, in addition to good results of this treatment, are reported. The mechanism of pain relief obtained through surgical correction of nasal septum and turbinate is discussed. These data make us conclude that there are multiple etiologic factors involved, which makes us question the fundamental role of the mechanical aspect.

## INTRODUCTION

Headache is a very frequent stimulated symptom, which is the complaint of half of the subjects that come to the physician ^1^. It is also a difficult to study symptom owing to the great variety of clinical and etiological presentations ^2^. In general, people with headache and craniofacial pain of difficult treatment and long evolution come to the ENT, after they had been investigated by the general practitioner, neurologist and ophthalmologist ^3^.

As to nasal causes of headache, in addition to inflammatory, allergic and neoplastic etiology, such as sinusopathy, polyposis, specific or allergic rhinitis, abscesses and tumors ^4^, we should also consider the cases in which anatomical variations of the nasal cavity would be enough to determine the painful symptom. The ENT becomes more responsible for the investigation, not limited to excluding evident diagnoses, but attaining to the particularities of the nasal cavity anatomy, making use of computed tomography (CT) and nasosinusal endoscopy ^5^. It is noted, though, that many factors may be implied in the production of pain of nasal etiology, so as that the pathophysiological aspect that relates nasal anatomical variations and craniofacial pain becomes more important. Many authors have studied the topic since the beginning of the century, defining clinical syndromes such as anterior ethmoid nerve syndrome ^6^, nasociliary nerve syndrome, olfactory tissue syndrome ^7^, septal contact headache ^4^, four-finger headache syndrome ^8^ and nasal spore headache ^9^. Many clinical observations made by these authors, including the good results of treatment of these patients with simple clinical procedures, are relevant in the determination of the cause-relation between nasal anatomical variations and craniofacial pain.

Conversely, we frequently observe the gross deformities of nasal cavities in asymptomatic patients, according to the reports ^4^, because not all patients with impaction, referring to septal spores in contact with the lateral wall, have headache.

The objective of the present study was to discuss the many different pathophysiological aspects that relate anatomical abnormalities of the nasal cavity with craniofacial pain, as well as clinical and therapeutic implications.

## LITERATURE REVIEW

### Anatomy

The causal relation between nasal anatomical variations and headache became the object of investigation of many different authors that started to give special attention to the role of sensorial inervation of the nasal cavity, because its stimulation, specially mechanical, would be considered the triggering of the referred pain 6, 4, 8, 9, 10.

Ophthalmic and maxillary branches of the trigeminal nerve are responsible for nasal mucosa sensitivity. The ophthalmic branch is divided into the following nerves: lachrymal, frontal and nasociliary, and in turn it originates anterior and posterior ethmoid and intratroclear nerve. Behind these branches, the ophthalmic nerve is responsible for the sensitivity of the ocular globe, palpebra, forehead, root and lateral portion of the nasal pyramid, ethmoid cells and nasal mucosa of the medium and posterior conchae, and corresponding region to the nasal septum ^7^.

The anterior ethmoid nerve is the pathway responsible for the sensitivity in the nasal region considered to trigger the pain, and its pathway is described as follows: it leaves the orbit through the foramen and anterior ethmoid canal to enter into the anterior cranial fossa; after passing through the dura and bone, it reaches the nasal cavity through the fissure close to crista galli. The nerve goes down through the sulcus on the internal aspect of the nose bone and then is exteriorized by going through the nose bone and superior lateral cartilage as external nasal nerve. In the nasal cavity, there are medial branches to the septum and lateral branches to the anterior portions of the medium and superior concha and to the lateral wall anterior to them ^6^. The anatomical characteristics of the anterior ethmoid nerve, that is, its superficial pathway concerning the nasal mucosa and the narrow bone canals that it crosses, makes it susceptible to pathological processes ^7^.

The maxillary branch, through pterygopalatine nerves, sends posterior and superior nasal branches, responsible for the sensitivity of medium and superior conchae and upper meatus. Posterior-inferior nasal branches, coming from the major palatine nerve, also a branch of the maxillary nerve, are responsible for the floor of the nasal cavity, inferior and medium meatus, in addition to inferior concha. The superior region of the septum has the sensitivity determined by the nasopalatine nerve, another branch of the major palatine nerve, which heads to the incisor foramen. Moreover, the maxillary nerve fibers, when exteriorized at the intra-orbital foramen through the intra-orbital nerve, send nasal branches to the skin of the lateral region of the pyramid, including the nasal ala. Finally, the higher area of the mucosa of the nasal cavity is inervated by maxillary nerve branches, except for the anterior portions of medium and superior conchae and the region anterior to them, as well as the area corresponding to the nasal septum, which are inervated by the ophthalmic branches.

### Pathophysiology

The sensitivity of the nasal mucosa was investigated through stimulation with faradic current in many different areas of the nasal cavity of volunteers that had to describe what they felt. It was observed that conchae and ostia are much more sensitive to stimulation than paranasal sinuses recovering mucosa ^12^. Thus, it was determined that the headache of sinusopathy has nasal origin, and not from the infected paranasal sinuses, as we could have expected. The same authors were supported by the observation that they had more intense pain caused in their experiments, in the presence of congestion and hyperemia of the concha, compared to the moment they were free from such affections, regardless of the paranasal sinuses status. They were also supported by the suspension of pain in patients with sinusitis by retracting or anesthetizing the conchae and nasal structures. It was also observed that the referred pain affected areas of the maxillary branch and fewer areas of the ophthalmic branch. The study was used as a reference in subsequent studies ^7^. Other authors that caused chemical and tactile stimuli (pressure) in different regions of the nasal cavity, detected the onset of referred pain both in the regions inervated by the ophthalmic branch and by the maxillary branch of trigeminal nerve ^3^. They also defined the inervation of the anterior portion of the medium concha and the corresponding region of the septum by the anterior ethmoid nerve and upon stimulating these areas, they detected pain in the distribution of infra and supra-troclear nerves, skin divisions of the ophthalmic branch, corresponding to inner canthus and supraorbital region. Some observations consider that the triggering stimulus of symptoms occurs in the area named retro-tubercular, located behind the septal thickness at the level of the chrondro-cutaneous joint, which is distributed from the ophthalmic branch ^7^. However, other authors consider that this region has inervation by the sphenopalatine ganglion ^6^, that is, maxillary nerve.

The importance of the sensorial inervation of the nasal mucosa in triggering the painful symptom was reinforced by the relief of pain after application of topical anesthesia, cocaine, in the nasal cavity, specifically between the medium concha and the septum, whose sensitivity is conducted by the anterior ethmoid nerve 3, 8, 10, 13. Vasoconstrictors ^9^ and lidocaine ^13^, that cause immediate relief, characterize the positive response to the test, whose clinical meaning will be shown later.

These data support the idea that we can define areas of the nasal mucosa that trigger pain such as the anterior portion of the medium concha and the corresponding region of the nasal septum 3, 4, 6.

The pain referred by patients is placed specifically in the areas supplied by the skin branch of the ophthalmic nerve, especially supra and infra-troclear nerves, that is, internal canthi, supra-orbital region and temporal-zygomatic region 3, 9. There are authors that also include the neck and upper limbs ^6^.

The mechanism of referred pain was quoted in 1946 ^7^, stating that the association of nasal and intraorbital sensitive fibers and their nuclei at the central level explained the similarity of the symptoms of the pathological involvement of both areas, showing that the orbital pain may be produced in intra-nasal regions. Afferent fibers of pain receptors located in the nasal and paranasal mucosa determine the same pool of sensorial neurons in the nucleus of the trigeminal nerve than fibers coming from skin receptors. These two pathways end up in the same neurons of the common cortical area. “The cortical center can not differentiate the original peripheral source of impulses in this common pathway, thus, when the mucosa is stimulated, pain afferent impulses are falsely located after they reach the sensorial cortex. They are poorly interpreted and based in previous experienced such as coming from the skin, region from where the impulses normally reach this point in the brain” ^5^. Another mechanism of referred pain considers that the presence of neurogenic edema in distant regions of the stimulated site: trigeminal fibers that contain substance P, upon stimulation, can trigger antidromic impulses responsible for the release of substance P in other areas innervated by the trigeminal branches, which leads to an inflammatory process in these sites, explaining the onset of pain in the distant region of the stimulated area 14.

It was considered that the stimulus of the reported nasal mucosa region was caused by the contact between the subsequent nasal structures of anatomical variations of constitutional or traumatic origin 4, 8, 15, medium concha hypertrophy or pneumatization 3, 11, 14, 16.

Medium conchae pneumatization may occur because they are part of the ethmoid complex that expands according to potential spaces ^17^.

A long list of anatomical variations that predispose to headache, affecting the nasal septum, agger nasi cells, medium concha, ethmoid bulla and combination of them all, is considered in this pathophysiology ^5^, including the affections to superior concha, asymmetry of ethmoid complex and skull base affections ^10^.

Other authors have carried out a review of a series of coronal sections of computed tomography (CT) of paranasal sinuses, analyzing bone affections, including medium concha pneumatization, agger nasi cells and Haller cells, and did not find clear correlation between these affections and the pathology ^18^.

Many authors consider the existence of pressure between these structures as the main stimulus 3, 4, 6, 9, 10, 11, but even without permanent contact between the structures, it is defined according to the nasal cycle, and is influenced by physical, climatic stimuli, such as moisture and temperature, in addition to chemical, allergic and inflammatory stimuli 10, 11.

The contact between the structures, in addition to being a mechanical stimulus in those regions considered as origin of the pain, promote local inflammatory process owing to mucociliary dysfunction, which takes to release of mediators that are related with the painful process. Moreover, the approximation of mucous surfaces leads to dryness of the mucosa because of Bernoulli’s effect (increase in speed of flow and reduction in pressure, according to reduction of the section area) in airflow, with accumulation of mucus and limitation of ciliary function ^11^. Local inflammatory process helps to aggravate the pressure that exists between the structures by causing edema. Such affections create the conditions for the development of local infectious process, an important factor for onset of pain. The presence of mediators as P substance and histamine reduces pain threshold in the nasal mucosa receptors ^19^.

**Figure 1:**
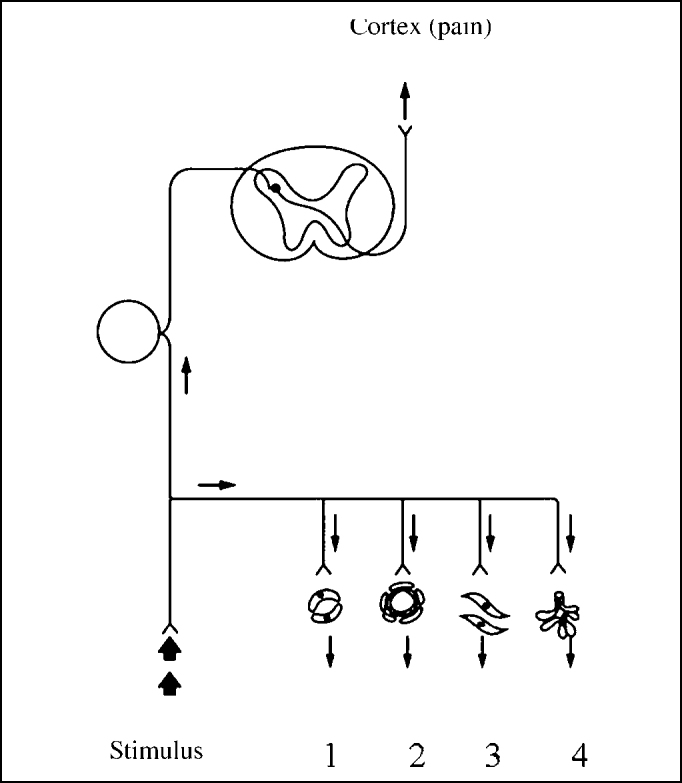
Axonal reflex showing the effects of substance P in the nasal mucosa through an antidromic impulse ^5^. (1- plasma leak, 2-vasodilatation, 3-smooth muscle contraction, 4-secretion).

The theory of the local reflex triggered by contact between structures, with release of vasoactive amines and onset of edema is a mechanism valued by the literature ^3^. This mechanism can be the substance P as a mediator of the reflex. P substance is a neuropeptide known since 1931 and found in sensitive nervous fibers of the nasal and paranasal mucosa, among other sites 20, 21. Different stimuli in polymodal receptors located in the nasal mucosa, such as infectious, chemical, caloric or simply mechanical (pressure) irritating agents may generate an ortodromic impulse to the cerebral cortex, mediated by substance P, responsible for the painful stimulus. In addition to ortodromic impulse, such stimuli generate also antidromic impulses, that is, contrary to what we could expect from afferent fibers, capable of releasing P substance in the nasal mucosa, mediating plasma leak, vasodilation, smooth muscle contraction and hypersecretion. This mechanism is called axonal reflex (figure 1). Mucosa edema may increase the existing pressure among the structures, maintaining the process in a vicious cycle ^5^. The occurrence of local trauma by the contact and pressure between the structures can also lead to release of substance P in the nasal mucosa ^14^.

Another mechanism related with the reported anatomical variations is the obstruction of drainage ostia of the paranasal sinuses, resulting in poor aeration, leading to vacuum headache or hypoxia 5, 10, 15, 22.

In addition to these mechanisms, headache is reported as a secondary symptom to nasal obstruction owing to septum deviation in rates that range from 23% ^23^ to 58% ^15^, and its surgical correction would lead not only to improvement of nasal obstruction, but also headache ^24^. Alternatively, it is also observed that failure in surgical treatment to control pain may be related to persistence, at least partial, of nasal obstruction ^25^.

Authors in a Brazilian study have observed nasal obstruction in 82% of the 11 cases of medium turbinate headache syndrome ^26^ .

There is the consideration that anatomical variations causing narrowing of nasal cavities would represent the triggering factor or the first stage of different forms of headaches ^10^. Such affections cause edema of nasal mucosa when submitted to climatic or hormonal affections and consequently to pressure between the structures and hypoventilation of paranasal sinuses, leading to tissue hypoxia and serotonin release and other vasoactive substances, finally inducing to crisis. The same authors demonstrated the value of nasal surgical treatment to relieve such cases.

The population affected with nasal obstruction by septum deviation and headache from different clinical modalities was studied and submitted to surgical treatment and among the eleven cases in which there was complete elimination of symptoms, four had diagnosis of headache ^24^.

Among the different pathophysiological mechanisms of headache, the role of traumatic nasal deformities is valued by some authors that consider that they are aggravated by climatic changes ^27^.

Other pathophysiological correlation is reported through 2 cases in which the patients had clinical diagnosis of headache in episodes, whose investigation showed sinusopathy ipsilateral to pain and whose treatment made symptoms cease. The explanation would result from connections between trigeminal nervous fibers, responsible for inervation of paranasal sinuses with parasympathetic neurons of sphenopalatine ganglion ^28^.

The fact that trigeminal fibers are widely distributed around important vessels of the central nervous system, comprising a trigeminal-vascular system, reveals the pathophysiological role of these fibers. When stimulated, they would lead to an inflammatory process of these vessels, mediated by substance P and gene-related to calcitonin, which is experimentally observed in dura of animals by plasma leak, activation and degranulation of mast cells and increase in vesicles of endothelial cells, which could trigger headache crises ^29^.

### Clinical and Diagnostic Aspects

From a clinical perspective the literature brings many symptomatic descriptions and definitions of syndromes related with nasal anatomical causes and headache and craniofacial pains, such as anterior ethmoid nerve syndrome ^6^, where the patients complains of frontal pain, right or left, extending somewhat above the supra-ciliary line and a little below the nasal bones, sometimes up to the nose tip, which may include the orbit. Pressure pain, less localized, in the ophthalmic nerve area, ipsilateral nasal congestion and posterior rhinorrhea form the olfactory fissure syndrome ^7^. As to type and intensity of pain there are descriptions of intermittent pain 3, 8, with pressure characteristics 15, 22, pulsatile or sharp ^9^, severe and disabling 9, 22, and many authors consider it mild to moderate ^3^. As to location, inner canthus, supraorbital and frontal region 3, 8, 9, 15, 22 and it may reach the orbit ^9^, infraorbital region and temporal-zygomatic region 8, 22, both unilateral ^3^ and bilateral, when it forms the four-finger syndrome, in an allusion to the position taken by the victims of these symptoms 8, 22. Other regions such as the vertex and the nape are also quoted ^22^. The painful episodes normally last some hours 3, 8, with frequency varying from daily 9, 22 to weekly or rare ^15^.The presentation may be related with level of nasal mucosa congestion, influenced by the position of the patient by the action of gravity 3, 8, 11, 24, and also by external stimuli, such as climatic affections ^10^. Nasal complaints are observed following the painful presentation 3, 10, 14. The presence of visual and digestive aura before the onset of pain is also reported ^10^. Normally there is no improvement with analgesics or treatment for migraine ^3^.

The physical examination of these patients that present nasal anatomical variations such as the ones reported before is based on diagnostic suspicion, but many times the anatomy is considered normal, with absence of suggestive signals of inflammatory or infections process ^3^.

Such patients are diagnosed as having the pathology based on clinical data, according to the different characteristics presented above, and exclusion diagnosis has to rule out sinusal infectious, neurological and ophthalmic causes, and vascular and episodic headache, according to the report 3, 9, 11, 22. Therefore, for the diagnosis, such authors report the need of neurological assessment and investigation, including the attempt to treat, as well as by an ophthalmologist. Some authors do not exclude vascular and episodic headaches, because they consider they are triggered by nasal causes ^10^, such as those reported before.

The conduction of CT scan is important to rule out the affection of paranasal sinuses, considering the exclusion perspective, and it can also visualize subtle anatomical variations of nasal cavities, normally not detected in the physical examination 3, 5, 11, 14.

Similarly, naso-sinusal endoscopy is a useful diagnostic resource because it also allows visualization of the most difficult to access regions of the nasal cavity 3, 5, 14.

The application of anesthetic or vasoconstrictive medication in the nasal cavity, especially between the medium concha and the septum, carried out during a painful episode, is the test with topical medication, or test with anesthetic, a resource frequently used to confirm the pathophysiological role of nasal anatomical variations in the determination of the reported clinical picture. Once the current pain is relived, the test is considered positive, confirming the diagnosis of the entity in the opinion of many authors 3, 4, 5, 8, 10, 11. They use cocaine for the tests, whereas others use lidocaine 13, 16 and topical vasoconstrictors ^9^.

### Therapeutic aspects

Patients that suffer from painful episodes whose cause in nasal anatomical variations may be clinically treated aiming at relieving the contact and pressure in the structures by reducing mucosa edema using systemic and topical decongestants, topical steroids, antibiotics and immunotherapy 3, 13.

Surgical treatment is indicated based on failure of clinical treatment 3, 5, 25 whose suggested duration would be six weeks to two months ^13^. In addition to failure in clinical treatment, the surgical indication is based on the anesthetic tests, because it gives the prognosis of the surgical treatment considered, because if there is symptomatic relief with the application of the anesthetic (or even decongestant), the surgery would be better indicated 5, 8, 10. The use of placebo (sterile solution) rather than anesthetic medication is also suggested and in the case it promoted pain relief, it would contraindicated the surgery, because it would predict a high likelihood of recurrence ^4^.

The procedures reported include septoplasty, associated or not with partial or total medial turbinectomy, septal submucous resection 3, 4, 8, 10, 16, 26, rhinoseptoplasty ^24^, endoscopic medial turbinectomy^10^, ^14^ and sphenoidectomy ^10^. There is reference to avulsion of ethmoidal nerves for difficult to treat cases ^6^.

Patients are followed up after the surgery to observe the results for at least two months ^8^ up to seven years ^10^ (Table 1).

**Table 1:** Follow-up after surgical treatment to observe results.

**FOLLOW-UP DURATION**	**AUTHOR**	**MEAN (MONTHS)**
2 months to 42 months	Morgestein & Krieger, 1980	22
8 months to 7 years	Novak, 1984	46
12 months	Schonsted-Madsen et al., 1986	12
2 years to 6 years	Hoover, 1987	48
4 months to 48 months	Low & Willatt, 1992	18
2 months to 24 months	Goldsmith et al., 1993	13
12 months	Clerico & Fieldman, 1994	12
6 months to 16 months	El-Simily, 1995	11
18 months	Kamal, 1995	18
10 months to 52 months	Pereira, 200	30

Patients are considered cured or with significant improvement of algesic episodes as a result of surgical treatment in 63.6% ^15^ to 100% 3, 13 of the cases (Table 2).

**Table 2:** Percentage of patients considered cured or with significant improvement after surgical treatment according to studies carried out by many authors.

%	AUTHOR
89	Morgenstein & Krieger, 1980
0	Peacock, 1981
98	Novak, 1984
77	Schonsted-Madsen et al., 1986
100	Goldsmith et al., 1993
100	El-Simily, 1995
99	Kamal, 1995
69,2	Koch-Henriksen et al., 1984
63,6	Low & Wilatt, 1992
78,5	Novak & Marek, 1994
80,95	Wilkmann et al., 2000
54	Pereira et al., 2000

There is a retrospective study of 913 patients treated of nasal obstruction caused by septal deviation in which the authors observed the frequency of 23% of patients with facial pain associated with nasal obstruction, which were reduced to 22.2% after submucous resection of the septum ^23^. Other report described the reduction of frequency of symptomatic relief after one year of follow-up (mean of 29.1 months) when compared to less than one year (mean of 7.3 months) from 79.3% to 46.2% ^15^.

## DISCUSSION

Craniofacial pain and headache are very frequent complaints ^1^, which are difficult to study symptoms because of the wide variety of clinical presentations and little objectivity obtained with its assessment. The multiplicity of etiologies is another important factor. Among them, the psychosomatic cause is defined as diagnosis, which may limit a more detailed investigation in search for a solution to the patient. Naso-sinusal etiology or rhinogenic headache is a known cause of pain. Nasal and paranasal structures take a large territory of the face and expose their vast mucosa surface to environmental affections. Acute sinusitis, intranasal tumors, septal hematoma or abscess, allergic rhinitis and headache by septal contact are considered causes of rhinogenic headache ^4^. There is no question that acute sinusitis or the pressure generated by the tumor may cause pain, as well as headache frequently associated with allergic rhinitis episodes. However, the correlation between nasal septum deformity and craniofacial pain is a subject that is little explored.

Both nasal anatomical variations and headache and craniofacial pain are very frequent; authors started to consider the existence of a cause relation between them, suggesting clinical syndrome and simple surgical treatment approached.

Major emphasis is placed on the anatomical aspects of the nasal cavity, which present frequent variations including septal deviations, forming spores or not, hypertrophy, pneumatization or other concha affections and variations of the lateral wall nasal cavity structures. These variations promote an approximation of the structures and even contact between them. Many authors have seen in it as cause of craniofacial pain, which is described as the presence of pressure between the structures 3, 4, 6, 8, 9, 10, 11.

A relevant comment is necessary about the pressure placed by the pneumatized medium concha. Pneumatization of the concha is caused by the ethmoidal cells according to the presence of space in the nasal cavity ^17^. Under this perspective, it is hard to thing that the pneumatized medium concha that makes pressure over the septum, that is the same that occupied the space, has interrupted the stimulus to continue the expansion, ceasing the pressure. However, it is interesting to observe that the medium concha on the stenosis side does not probably have enough space to pneumatize and even so, we observe many times the presence of bilateral extensive pneumatization of the medium concha, even without nasal septal deformities, suggesting that they could be primary and pathological, but the literature has almost no explanation about this fact.

It is observed that the anatomical aspect, generating mechanical stimulus, is considered the main pathophysiological factor and when some authors define the clinical syndromes, they conceptualize the need to exclude the naso-sinusal process 6, 14. Experimental observations were the main evidences that based this concept^8^, ^12^. After all, once we stimulate the nasal mucosa, be it through faradic current, chemical substances or pressure, it causes pain on the back on the regions of ophthalmic and maxillary nerve distribution. The medium concha portion and the area that corresponds to nasal septum would be especially implicated and the variations affecting this region, especially septum deviations and hypertrophy or medium concha hypertrophy or pneumatization, are very valued as a cause of pain. Such region has its inervation through the anterior ethmoidal nerve, branch of the ophthalmic nerve ^30^. This nerve has a superficial pathway that crosses the bone channels and ends as an external basal nerve, of cutaneous distribution. Such characteristics make the branches exposed to pathological processes.

Another evidence of the anatomical role, by mechanical stimulus and especially of the reported region, is the realization of pain relief when applying in this region an anesthetic or vasoconstriction substance 3, 8, 10, 13.

Intranasal mechanical stimulus causes distant symptoms, affecting the area of ophthalmic and maxillary distribution regions by the mechanism of referred pain, that is, afferent fibers coming from the nasal mucosa end in central areas close to those coming from the cutaneous regions, which the cortex may interpret as the stimulus coming from those regions ^5^.

We can observe in this study that there are other factors involved when we have nasal anatomical variation that causes contact between the structures and/or narrowing of the nasal cavity, which may be related with pain onset. Among them, we include mucociliary function, with accumulation of mucus, creating an environment prone to the development of an infectious process 11, 15 and logically with the release of inflammatory mediators capable of generating the painful symptom.

The presence of anatomical variations can also cause obstruction of the paranasal sinuses ostia drainage, which because of hypoventilation and hypoxia, may also cause pain 5, 10, 15, 22. These two factors demonstrate that the infectious aspect cannot be disregarded, and a sinusopathy is likely, considering that we have the necessary ingredients, such as mucociliary dysfunction and ostium obstruction in critical areas. We can consider that contrarily to excluding phenomena, anatomical and infectious factors are interconnected.

We could also argue that the exclusion of sinusitis for the diagnosis of headaches caused by nasal anatomical variations would be made in fact by lack of appropriate diagnosis. In the 60’s, when studies about the topic were collected ^6^, some authors defended this concept in a period in which the sensitivity of the diagnostic methods were lower than more recently. Even so, this concept became more intense, and it is likely that the sinusitis that affect ethmoidal cells or small thickness of the frontal and maxillary sinus mucosa would be left undetected and the patient would have the diagnosis of headache by anatomical cause, excluded from infectious sinusal cause. Currently, such patients could make the diagnosis of sinusitis through CT scan. Conversely, an analysis of the coronal sections by CT scan of patients with and without history of sinusitis has led to the observation that this exam could present false positive diagnosis, once we found minor mucosa affections in asymptomatic patients, concluding that the diagnosis should be based on individual assessment of each case ^18^. However, other authors listed some anatomical variations found in the CT scan considered to be significant predisposing factors both of headache and sinusopathy ^5^.

Climatic abnormalities of humidity and temperature, chemical, allergic and hormonal stimuli are reported as causes of nasal mucosa edema, especially in narrow regions, which may cause the contact between the structures 10, 22. We may speculate, however, that the nasal inflammatory process may be sometimes independent of the anatomical variations or contact between the mucosa, which may be aggravated by it.

The participation of neurogenic edema, through the action of mediators released by nervous sensorial fibers, for example, P substance, should also be commented. Stimuli for its triggering are mechanical, chemical, infectious and caloric ^5^.

The reach of this topic is expanded when nasal anatomical variations are considered triggered by some form of headache ^10^. The correlation between headache or migraine and etiology related with nasal and paranasal structures is interesting when we consider the trigeminal-vascular system, formed by the presence of trigeminal fibers around important central nervous system vessels, and when stimulated they produce inflammatory affections in these vessels, mediated by a peptide related with calcitonin gene (GGRP) and P substance, causing headaches ^29^. Trigeminal fibers are widely distributed in nasal and paranasal structures via ophthalmic and maxillary branches, exposing them to different stimuli. The trigeminal fiber network is capable, upon specific stimulation, of releasing distant mediators, via axonal reflex or antidromic impulse, such as the reported one. Therefore, we confirm that in the trigeminal vascular system there is an interesting pathophysiological explanation for the observations that relate nasal structures to headaches. Such correlation is observed in another way in the report of two cases with clinical diagnosis of headache in episodes, considered to be caused by ipsilateral sinusopathy ^28^. Trigeminal stimulation could be triggered by parasympathetic stimuli responsible for the symptomatology.

Regardless of the presence of nasal anatomical variations, little is said about the functional aspect, that is, the correlation between nasal obstruction per se and headache. We know that many authors consider headache as an associated symptom of nasal obstruction, and it is the secondary most common cause presented by these patients 15, 22, 23, 24. Regardless of the anatomical variation that causes obstruction, nasal poor ventilation and consequently complementary oral breathing lead to other pathophysiological mechanisms, such as absence of nasopulmonary reflex, with ventilation repercussion of pulmonary expansion, in addition to posture affections, reaching areas that go beyond the care provided by Otorhinolaryngologists. However, it is concluded that there is correlation between improvement of nasal obstruction and improvement of headache in patients submitted to nasal surgical treatment such as septoplasty and turbinectomy 15, 22, 24. What is the importance of each mechanism of symptom relief? Otorhinolaryngologists tend to locate the reasons for surgical procedures in nasal structures rather than in systemic mechanisms.

An important issue that has never been solved is the fact that we frequently observe in daily practice gross variations of nasal cavities, including concha deviations and hypertrophy, causing areas of contact between nasal structures and narrowing in patients that are completely asymptomatic from a perspective of facial pain or headache. It makes us wonder about the mechanisms we referred to - infectious, effect of climatic changes, neurogenic edema and migraine correlations, which may have effects that vary individually in each case. It is reasonable to think that these mechanisms occur also in the absence of anatomical variations, which are aggravating. The clinical representation of these phenomena should therefore range from subject to subject. It hinders the definition of nasal etiology. We shall not consider an anatomical variation to be solely responsible for a manifestation, nor conversely, exclude the etiology in a patient who has normal nasal cavities. Pathogenicity of different bone affections (agger nasi cells, Haller cells and concha bullosa) found in a review of CT scan sections is questioned, because these findings are frequent in asymptomatic patients ^18^.

Therefore, it is realized that there are many factors related with production of pain with nasal etiology. The argument that anesthesia of the nasal cavities would confirm the mechanical pathophysiological role of anatomical affections does not consider these multiple factors. We may speculate that the application of an anesthetic in regions of the nasal cavity causing relief of painful symptoms, would confirm the role of sensorial inervation of nasal mucosa, but it does not clearly define which pathophysiological mechanism is the most important one. It is timely to mention that there are reports of vasoconstriction in the test and upon retracting the mucosa, they would exclude the structures by eliminating the contact and consequently the mechanical stimulus for pain ^9^. This test is more specific for anatomical affections than those that use anesthetic, but they also cause physiological affections that do not consider it as an objective. It is not known to what extent the relief of pain caused as nasal decongestion when applying the vasoconstrictor has an impact in the subjective sensation of pain.

As to validity of this test, we could propose its conduction in subjects with non-rhinogenic headache, so as to estimate its specificity to nasal causes.

The clinical picture designed by nasal affections would basically correspond to pain, with variations of these characteristics, depending on the author, associated with the observation of some anatomical affection in the physical examination of the nasal cavities, which may cause suspicion of causal relation. The main difference in the description of the clinical picture consists of a group of authors that exclude vascular headache characteristics, considering differential diagnosis 3, 8, 9, 11, 14, 15, 22, and others that include them ^10^.

The diagnosis of a presentation of craniofacial pain related with nasal anatomical variations is based on clinical suspicion, that is, history and physical examination. The affections to physical examination may be subtle, being necessary to assess them by nasosinusal endoscopy CT scan 3, 4, 5, 8, 10, 11, 22. The test with anesthetics is very valuable for the diagnosis 4, 5, 8, 11, but there are authors that see limitations in the test, such as practical difficulties to apply it in the presence of pain and the fact that it is not applied with scientific basis. As mentioned before, the test would demonstrate neuronal implications, that is, sensorial inervation, which does not confirm the mechanical key role.

Another important aspect to be considered is the treatment suggested by the authors when we define the nasal anatomical etiology. The clinical treatment is mentioned but with unsatisfactory results 3, 13. Surgical treatment is emphasized, because as we saw it, the anatomical role is very valuable in pathophysiology. Its indication is based on failure of clinical treatment 3, 5 and in the conduction of the test with anesthetics, which would predict surgical results 5, 8, 10. However, the use of placebo instead of anesthetic drug is described and if there is pain cessation, the surgery is contraindicated ^4^.

The procedures focus mainly on nasal septum and medium concha: septoplasty, submucosa resection, partial or total turbinectomy, and also rhinoplasty. Curiously, some authors report ethmoidectomy and sphenoidectomy as treatment 3, 10, 14, but they do not convincingly clarify the criteria for such indications, which seem to be conflicting with the concept defended by some of these authors ^3^ that sinusopathy is a differential diagnosis, and that is should be excluded so that we can define the nasal etiology owing to anatomical affections.

Regardless of the employed procedures, all authors reached good results in their samples, with total relief or significant improvement of pain in most of the operated patients: 63.6% ^15^ to 100% 3, 13 up to the moment they were followed up. Follow up varied from 12 months^22^, ^14^ to 46 months on average ^10^ within the ten studies that showed these figures. Factors related with post-surgical clinical improvement are studied in an interesting survey, which included follow up shorter than one year related with good outcomes, observing a higher rate of recurrences after this period ^15^. This observation makes us think about the mechanism of symptomatic relief generated by surgical procedures. To some extent, they cause damage to the nervous branches implied in the pathophysiology, that is, branches of ophthalmic and maxillary nerves distributed in the nasal mucosa, and these are difficult to quantify lesions, but certainly they have repercussion on the conduction of sensorial stimuli in this region. This fact could theoretically support the minimization of painful symptoms after surgical treatment. Late follow up is considered essential in the conclusion of surgical treatment efficacy by authors in Brazil ^26^, which are followed up for up to 4 years, considering the surgical treatment as effective.

The role of nervous lesion in the symptomatic relied may be historically exemplified in the indication of anterior ethmoidal nerve sectioning via transorbital access and also by avulsion of the anterior and posterior ethmoidal nerves by abolition of the painful presentation in cases of difficult treatment ^7^.

If this mechanism was considered predominant for the impact of surgical result, it would take a potential of major recurrence, depending on the regeneration of damaged fibers, which would be in agreement with the study reported that correlated the best results in short-term follow up ^15^. It is worth mentioning the illustration of patients submitted to rhinoplasty, which as a result of time, recovers gradually his skin sensitivity. We should not disregard the placebo effect that surgical procedures have on patients, once it is a matter of surgical procedures, but no double blind studies were performed. This aspect is reported in one study but the author did not consider that the placebo effect could last for over one year, and since good results were found beyond this period, it is believed to be due to surgical treatment ^24^.

## CLOSING REMARKS

The correlation between headache and craniofacial pain with nasal anatomical aspects, from a pathophysiological aspect, is based on the mechanism of referred pain that includes the territories of the ophthalmic and maxillary trigeminal nerves. Triggering stimuli, however, should take into account the mechanical factor that is intimately related with inflammatory and infectious factors. Complex mechanisms such as neurogenic inflammation, correlation with migraine and nasal obstruction are involved. Surgical treatment, in addition to anatomical correction, may influence the sensitivity of the nasal mucosa and its efficacy depends on long term follow up in addition to the fact that placebo effect many be involved.
